# Preoperative Screening and Case Cancellation in Cocaine-Abusing Veterans Scheduled for Elective Surgery

**DOI:** 10.1155/2013/149892

**Published:** 2013-08-28

**Authors:** Nabil Elkassabany, Rebecca M. Speck, David Oslin, Mary Hawn, Khan Chaichana, John Sum-Ping, Jorge Sepulveda, Mary Whitley, Yasser Sakawi

**Affiliations:** ^1^Department of Anesthesiology, Philadelphia VAMC, 3900 Woodland Avenue, Philadelphia, PA 19104, USA; ^2^Department of Anesthesiology and Critical Care, Perelman School of Medicine, University of Pennsylvania, 3400 Spruce Street, Dulles 6, Philadelphia, PA 19104, USA; ^3^Department of Behavioral Health, Philadelphia VAMC, Philadelphia, PA 19104, USA; ^4^Department of Surgery, Birmingham VAMC, Birmingham, AL 35249-6810, USA; ^5^Department of Anesthesiology, Dallas VA Medical Center, Dallas, TX 75216, USA; ^6^Department of Clinical Pathology, Philadelphia VAMC, Philadelphia, PA 19104, USA; ^7^Department of Anesthesiology, Birmingham VA Medical Center, USA

## Abstract

*Background*. Perioperative management of cocaine-abusing patients scheduled for elective surgery varies widely based on individual anecdotes and personal experience. *Methods*. Chiefs of the anesthesia departments in the Veterans Affairs (VA) health system were surveyed to estimate how often they encounter surgical patients with cocaine use. Respondents were asked about their screening criteria, timing of screening, action resulting from positive screening, and if they have a formal policy for management of these patients. Interest in the development of VA guidelines for the perioperative management of patients with a history of cocaine use was also queried. *Results*. 172 VA anesthesia departments' chiefs were surveyed. Response rate was 62%. Over half of the facilities see cocaine-abusing patients at least once a week (52%). Two thirds of respondents canceled or delayed patients with a positive screen regardless of clinical symptoms. Only eleven facilities (10.6%) have a formal policy. The majority of facilities (80%) thought that having formal guidelines for perioperative management of cocaine-abusing patients would be helpful to some extent. Results. 172 VA anesthesia departments' chiefs were surveyed. Response rate was 62%. Over half of the facilities see cocaine-abusing patients at least once a week (52%). Two thirds of respondents canceled or delayed patients with a positive screen regardless of clinical symptoms. Only eleven facilities (10.6%) have a formal policy. The majority of facilities (80%) thought that having formal guidelines for perioperative management of cocaine-abusing patients would be helpful to some extent. *Conclusions*. There is a general consensus that formal guidelines would be helpful. Further studies are needed to help formulate evidence-based guidelines for managing patients screening positive for cocaine prior to elective surgery.

## 1. Introduction

The National Survey on Drug Use and Health (NSDUH) estimates that 5 million Americans are regular users of cocaine, with 6000 new users daily, and more than 30 million have tried cocaine at least once [1]. Combined data from 2004 to 2006 indicate that an annual average of  7.1% of veterans met criteria for a past year substance abuse disorder [1, 2]. More specifically, 14.1% of respondents who served in the military have a history of cocaine use [1]. 

Cocaine produces prolonged adrenergic stimulation by blocking the presynaptic uptake of sympathomimetic neurotransmitters, including norepinephrine, serotonin, and dopamine [3]. There are two chemical forms of cocaine: the water-soluble hydrochloride salt and the water-insoluble cocaine base (freebase or crack) [4]. Routes of administration include oral, intravenous, and intranasal [5]. Smoking the free base or crack cocaine results in very effective trans mucosal absorption and high plasma concentration of cocaine. It is metabolized by plasma and liver cholinesterases to water-soluble metabolites (primarily benzoylecgonine and ecgonine methyl ester (EME)), which are excreted in urine [6]. The serum half-life of cocaine is 45 to 90 minutes [5]. Thus cocaine can be detected in blood or urine only several hours after its use. However, its metabolites can be detected in urine for 3–7 days after ingestion and up to 16 days in chronic heavy users [7]. Acute cocaine toxicity results in cardiovascular [8], respiratory [9], and central nervous system effects [10, 11]. Cardiovascular effects include tachycardia, hypertension, prolonged QT interval, coronary vasospasm, and myocardial ischemia [12, 13]. Smoking crack cocaine can result in nonspecific respiratory symptoms and a picture similar to acute lung injury [14]. Central effects include the initial euphoria (cocaine high), psychomotor agitation, hyperthermia, seizures, intracerebral hemorrhage, and acute stroke [10]. Chronic effects of cocaine abuse include left ventricular hypertrophy [15], systolic dysfunction, dilated cardiomyopathy, diffuse alveolar damage, noncardiogenic pulmonary edema [16], and focal neurologic deficits [17].

Veterans with history of cocaine abuse presenting for elective surgery invoke a controversy for the anesthesiologist in whether to test for recent cocaine use, often delaying the procedure or to proceed without testing. The main concern is the increased risk of myocardial ischemia [8, 18], arrhythmia [12, 19], and stroke [10] in patients with acute cocaine toxicity. This clinical dilemma often leads to same day surgical case cancellations resulting in delay and/or denial of the surgical care needed for these veterans. Same day case cancellation also results in wasting of the operating room (OR) resources and of valuable health care dollars [20]. Currently, there are no published guidelines to support the clinical decision making regarding perioperative management of patients with history of cocaine abuse [21]. Clinical practices vary widely based on individual anecdotes and personal experience. Some physicians will routinely order a urine drug screen (UDS) the morning of surgery and subsequently delay or cancel a cocaine-positive patient due to the concern for intra-operative hemodynamic instability, myocardial ischemia, and/or acute cerebrovascular stroke. Other providers will proceed with elective surgery if the cocaine-positive patient does not exhibit clinical signs of toxicity. Triggers for screening patients for cocaine are also dependent on individual anesthesiologist's practice. The magnitude of this problem in the Veterans' Affairs (VA) health system is not well defined. 

The primary aim of this study was to identify the practices, procedures, and policies regarding cocaine drug screening and surgical case cancellation across VA facilities. The second aim was to determine the rate of same day case cancellation in two VA medical centers in Philadelphia, PA, and Birmingham, AL, due to positive cocaine urine drug screen.

## 2. Methods 

The Philadelphia Veterans Affairs Medical Center (PVAMC) Institutional Review Board (IRB) granted approval for this study protocol. A 7-item survey instrument was designed by the study investigators to survey the VA anesthesia departments' chiefs to identify practices, procedures, and policies of their departments regarding cocaine drug screening and surgical case cancellation due to positive testing. Following the initial questionnaire development, the survey instrument was reviewed by qualitative research experts from the Center of Health Equity and Research Promotion (CHERP) at the PVAMC, [R. M. Speck and J. Sepulveda] and from the Center for Surgical, Medical Acute care Research and Transitions (C-SMART) in Birmingham VA medical center [M. Hawn]. A draft of the survey was later sent to two VA anesthesia service chiefs [J. S. Ping and Y. Sakawi] and to the chief of behavioral health and addiction medicine at the PVAMC [D. Oslin] to give their feedback on the survey questions. The final survey instrument is included in the appendix. Respondents were asked to estimate the frequency of encounters with patients with a history of cocaine use in their departments and report if a formal policy exists for preoperative drug screening. Triggers for urine drug screen, timing of screening, action resulting from a positive drug test, and estimated cancellation rate due to positive drug screens were polled. Interest in the development of VA standards or guidelines for perioperative management of patient with a history of cocaine use was also queried. 

The survey was posted on the central anesthesia service sharepoint website for completion by the chiefs of anesthesiology services or their designees. Department chiefs were alerted via email to complete the survey. The e-mail notification included a printable version of the survey in case any facility preferred responding to a printed version of the survey to the central anesthesia service of the Veterans Health Administration (VHA). E-mail notification also went to each of the directors, chief medical officers, and medical center directors of the 21 Veterans Integrated Service Networks (VISN). For the purposes of this analysis, only responses from the anesthesia chiefs or their designees were included. Only one response from each facility was considered.

 A second aim of this study was to determine the rate of same day case cancellation among surgical patients due to positive drug screen in two VA facilities, namely, Philadelphia VAMC and Birmingham VAMC. Surgical case cancellation classified by reasons for cancellation was obtained for 2009 and 2010 by querying the Veterans Health Information Systems and Technology Architecture (VISTA) in both facilities. VISTA is an enterprise-wide information system built around an Electronic Medical Record (EMR), used throughout the VHA [22]. Patients' data were obtained through requests to the data warehouses of the corresponding VISN for each facility. To identify cocaine-related case cancellation, EMRs of patients whose surgery was cancelled on the same day of their scheduled procedure were reviewed. If there was a positive UDS for cocaine on the day of the cancelled procedure or a note from a physician (surgeon or anesthesiologist) in the medical record indicating the reason for cancellation as recent cocaine use, cases were designated as cocaine-related case cancellation.

### 2.1. Statistical Methods

Descriptive statistics, including frequencies, percentages, means, and standard deviations were calculated for the survey instrument and patient data. All analyses were completed with Stata version 11.0.

## 3. Results

**Figure 1:**
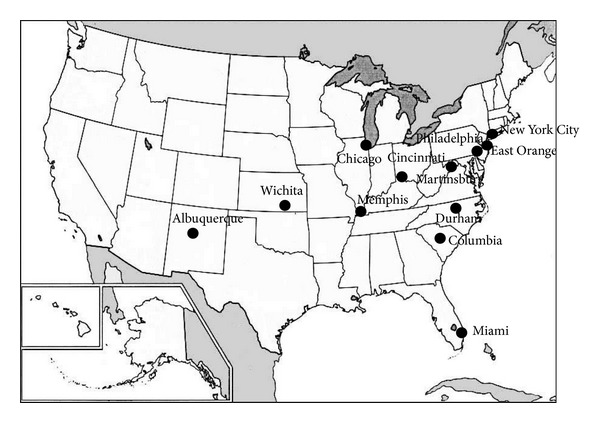
Map of U.S.A highlighting the cities that report the highest frequencies of surgical patients using cocaine.

The VA health system's 21 VISNs include 172 facilities with anesthesia departments. A total of 123 survey responses were received from 112 facilities, for a response rate of 65%. As stated previously, the analysis included only one response per facility. Eleven facilities had duplicate responses. The response from the anesthesia chief, or individual designated to function as the anesthesia chief, was considered for the analysis. The medical centers included in the analysis represented all 21 VISNs within the VHA.  Figure 1 displays a map of U.S.A highlighting the cities that report the highest frequencies of surgical patients using cocaine.

Over half the facilities reported that they encounter cocaine-abusing patients scheduled for elective surgery at least once a week (52%). Over one-third (36%) of respondents indicated their department sees patients with cocaine abuse problems 2 or more times a week. Fifteen facilities reported a daily encounter with cocaine abuse patients (13.6%), while 22% said they hardly ever do (less than once a month). 

The distribution of the reported criteria used to order a urine drug screen for cocaine was as follows; (1) combination of history of cocaine abuse determined by patients' self report or review of their EMR along with clinical suspicion of toxicity (42%), (2) clinical suspicion only (13%), (3) history of use determined from the patient or their chart (34%), and (4) never screen (11%). In terms of timing of the screening, one third (33%) of facilities screen during the morning of surgery, 8% screen during the preoperative visit, and another third (33%) screen during both the preoperative visit and the morning of surgery. 

**Figure 2:**
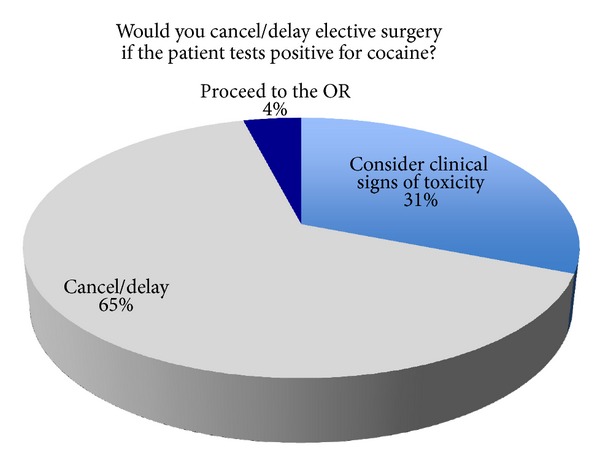
Percentages of anesthesia departments responses to a positive cocaine urine drug screen.

If a patient having an elective surgery tested positive for cocaine, 65% (*N* = 73) indicated they would cancel or delay the procedure regardless of the clinical signs and symptoms, only 4% would proceed to the OR; 31% of respondents indicated that clinical signs and symptoms of acute toxicity are also considered in addition to the results of the drug screen results when making the decision about cancelling procedures (Figure 2). 

When asked what percentage of practitioners in their department would cancel or delay an elective procedure for a cocaine-positive drug screen, the majority (59%) of the anesthesia departments' chiefs reported that all, 100%, of their providers would. Only 14% indicated that 50% or less of the providers in their department would cancel or delay the procedure for a cocaine-positive drug screen. The odds of having all of a department's providers cancel a case for a cocaine-positive urine drug screen were not influenced by department size, as quantified by the number of anesthesiologists (OR = 0.92, 95% CI = 0.83, 1.02) or certified registered nurse anesthetists (OR = 1.01, 95% CI = 0.91, 1.12). 

**Figure 3:**
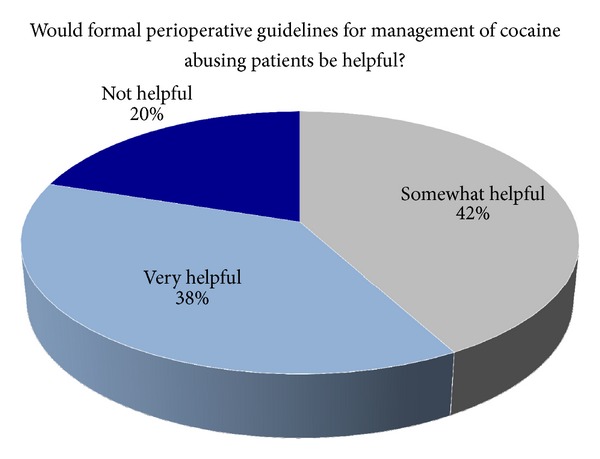
Distribution of the percentages of anesthesia departments finding perioperative guidelines for management of the cocaine abusing patients helpful.

Only 12% (*N* = 13) of respondents have a formal policy in place for preoperative screening of patients with active cocaine use. The odds of case cancellation were not significantly different between those who have a formal policy for perioperative management and those who do not (*P* = 0.1). Four fifths (80%) of respondents felt that VA standards or guidelines regarding screening for drug use in patients with a history of cocaine use undergoing elective surgery would be helpful, somewhat helpful (42%) to very useful (38%). The remaining one fifth (20%) indicated that standards or guidelines would not be helpful (Figure 3). 

In Philadelphia VAMC, the percentages of cases cancelled due to testing positive for cocaine were stable in 2009 (29 cases cancelled out of 438 cancellation for different reasons), and 2010 (27 cases cancelled out of 407), 7% and 6.8% respectively of all cancelled cases due to various reasons. In Birmingham, AL the percentage of cases cancelled due to positive cocaine drug screen was about 3.5% (14/389) of all cancelled cases in 2010. Both VA hospitals encountered patients with cocaine abuse scheduled for elective surgery twice a week or more. The Birmingham VAMC has a formal policy in place for perioperative management of these patients, while the Philadelphia VAMC does not. The rate of case cancellation due to positive UDS was significantly lower in Birmingham when compared with Philadelphia VAMC, 3.5% and 6.0%, respectively (*P* = 0.03). Whether this difference is due to the existing policy in place or just due to different attitudes among individual staff is unknown.

## 4. Discussion

Over half (52%) of the VA anesthesia departments encounter, at least once a week, cocaine abusing veterans scheduled for elective surgery. About two thirds of the VAs would cancel patients if they tested positive for cocaine (65%). Triggers for screening and timing of screening for cocaine were variable among different departments. Despite this relatively common problem, only 12% of the departments have a formal policy and/or guidelines for management of the cocaine abusing patients. The overwhelming majority (80%) of respondents to this survey indicate that guidelines for perioperative management of these patients would be helpful to some extent. The half-life of cocaine is about 90 minutes as it is rapidly metabolized by plasma esterases [23]. The UDS is testing for the presence of the cocaine metabolites, predominantly benzoylecgonine (BE) [4, 24]. The literature provides conflicting evidence as to whether some cocaine metabolites such as norcocaine or cocaethylene are equally potent to the parent drug, although it is generally accepted that BE is clinically non-toxic [6, 25–28]. Another point of controversy is whether patients with chronic cocaine abuse are more prone to the development of cardiovascular adverse events when compared with occasional or infrequent users [15]. Studies reporting cardiopulmonary adverse effects in the perioperative setting in patients who do not present with symptoms and signs of acute cocaine toxicity but still test positive on their UDS because of their recent use are very few and are mostly case reports [29–34]. Given the lack of strong evidence in the literature, management of these patients in the surgical setting is not standardized. In the current study, the majority of anesthesia departments (65%) would cancel patients who test positive for cocaine. Cancelling surgical procedures especially the morning of surgery is costly and results in inefficient utilization of operating room resources, as well as delay and often denial of treatment for this patient population. In the VA health system, the cost of case cancellation on the same day of surgery is $850 per cancelled case in 2006 dollars [35, 36]. In 2010 [37], the approximate cost for the cases cancelled due to positive UDS for cocaine will be $24,840 for Philadelphia VAMC and $12,880 for Birmingham VAMC. These numbers are only the tip of the iceberg. The actual cost per each facility is projected to be much higher because of the following reasons. First, for a case to conform to the definition of a cancelled case in VISTA, the case is cancelled on the same day of scheduled surgery and it has to be entered into the surgical scheduling system as of 9 a.m. the day before surgery [20]. It does not take into account any “add-on” case that shows up on the surgical schedule after that cut-off time. Also, this estimate does not take into account the amount of time lost during waiting for the test results before making a decision to proceed with surgery if the test was negative. This situation is especially important during the first case in the morning when there is no flexibility in changing the order of cases on the operating room schedule. The cost of the unused OR time in the VA has been estimated at $600 per hour in 2009 dollars [20]. These figures tend to be much higher in the private sector as the cost per cancelled case in the day of surgery ranges approximately between $1700 and $2025 [38, 39]. 

At Birmingham VAMC, the urine drug screen is ordered as part of the preadmission testing in the preanesthesia evaluation clinic if the patient has symptoms and signs of acute toxicity. In the morning of surgery, a positive urine drug screen for cocaine is not an indication by itself for cancelling the case. The patient has to show clinical signs of toxicity for the case to be cancelled.

A limitation of the current study includes the potential for recall bias as we sought the responses from the chiefs of the departments, and there was some room for estimation of the magnitude of the problem and speculation about the practice of individual anesthesiologists within the department. There is no reason to expect that this type of recall bias would be differential among various facilities. The average number of physicians in any VA hospital in this survey was 5.4 ± 4.2. The relatively small size departments may have helped decrease the margin of error in estimating the pattern of practice of practitioners in each department. Overall, the response rate for the survey was respectable (65%) and representative of all 21 VISNs of the VA health system. Another limitation of the current study is that the survey did not attempt to question whether the decision to cancel/delay surgical cases is dependent on the surgical case complexity, planned anesthetic, or the severity of symptoms and signs of clinical toxicity. Getting down to this level of detailed information would be more appropriate if the survey was directed to individual anesthesiologist rather than to the chiefs of every anesthesia department. 

Some studies have suggested the conditional safety of general anesthesia for elective surgery provided that certain conditions are met [40]. Safety of general anesthesia was conditional in the absence of clinical hemodynamic and behavioral signs and symptoms of acute cocaine toxicity and normal QT interval [40]. Other studies did not show difference in the outcome in the acute trauma setting of patients surgically treated in the first 24 hours after their trauma and subsequently during their ICU stay when they tested positive for cocaine [41, 42]. Barash et al. [43] studied the hemodynamic effect of intranasal cocaine in 18 patients undergoing coronary artery bypass surgery. The rise in the plasma cocaine level did not have any clinically significant sympathomimetic effect and appeared to be well tolerated in anesthetized patients with coronary artery disease. The current study sheds some light on the perioperative management of the cocaine-abusing patients within the VA health system. 

## 5. Conclusion

 Perioperative management of the cocaine-abusing patients varies between institutions, mainly based on culture of the institution and individual anecdotes. Same day case cancellation is costly and may be unjustified in some cases. There is a clear need for institution of guidelines or practice advisory for perioperative management of the cocaine-abusing patients. Education of the physicians about the evidence supporting or refuting the safety of general anesthesia in this setting is equally important.

## Appendix

### The Survey Questionnaire

*Dear VA anesthesia service chief*

The purpose of this survey is to identify current policies and practices within the VA health system related to screening patients for cocaine abuse before surgery. We appreciate the time you are taking to answer these questions; it is our hope that your answers will help us formulate future directives and guidelines related to this important clinical topic

Please provide the following information
VISN no:–––—
Facility name or number:––––––
City–––––
State–––—
How many practitioners in your department?
Anesthesiologist □ CRNAs □ Anesthesia Assistants (AA) □  
(1) How often does your department see patients with the cocaine abuse problem scheduled for elective surgery? (estimates are ok)
  □ Every day
  □ Twice a week
  □ Once a week
  □ Once every two weeks
  □ Once every month
  □ Hardly ever
(2) Do you have a formal policy for preoperative screening of patients for active cocaine use?
  □  Yes
  □  No, but we have a consensus agreement among our group
  □  No, it depends on each individual's practice (no consensus among our group)
(3) What are your screening criteria for cocaine use in patients in patients undergoing elective surgery ?
  □ History of use from patient or chart
  □ Clinical suspicion of toxicity
  □ We never screen
  □ Other, please comment
(4) When do you screen for cocaine?
  □ During the preoperative visit
  □ Morning of surgery
  □ Both
  □ We never screen
  □ Other, please comment
(5) If a patient having an elective surgery tests positive for cocaine, do you  cancel/delay the planned anesthesia/surgery?
  □ Cancel/Delay
  □ Proceed to the OR
(6) What percentage of practitioners in your department will cancel or delay an elective procedure for a cocaine-positive drug screen
  □ 10%
  □ 25%
  □ 30%
  □ 50%
  □ 75%
  □ 100%
(7) Do you think having VA standards or guidelines regarding screening for drug use in patients with a history of cocaine use undergoing elective surgery would be useful?
  □ Very useful
  □ Somewhat useful
  □ Not helpful
